# Effects of Structural Dimension Variation on the Vibration of MEMS Ring-Based Gyroscopes

**DOI:** 10.3390/mi12121483

**Published:** 2021-11-29

**Authors:** Zhipeng Ma, Xiaoli Chen, Xiaojun Jin, Yiming Jin, Xudong Zheng, Zhonghe Jin

**Affiliations:** 1 Micro-Satellite Research Center, Zhejiang University, Hangzhou 310007, China; mazhipeng@zju.edu.cn (Z.M.); xlchen@mail.nwpu.edu.cn (X.C.); jinyiming@zju.edu.cn (Y.J.); zhengxudong@zju.edu.cn (X.Z.); jinzh@zju.edu.cn (Z.J.); 2 Key Laboratory of Micro/Nano-Satellite Research, Hangzhou 310007, China

**Keywords:** ring gyroscopes, gyroscope modeling, fabrication imperfection, frequency split, geometrical compensation

## Abstract

This study investigated the effects of structural dimension variation arising from fabrication imperfections or active structural design on the vibration characteristics of a (100) single crystal silicon (SCS) ring-based Coriolis vibratory gyroscope. A mathematical model considering the geometrical irregularities and the anisotropy of Young’s modulus was developed via Lagrange’s equations for simulating the dynamical behavior of an imperfect ring-based gyroscope. The dynamical analyses are focused on the effects on the frequency split between two vibration modes of interest as well as the rotation of the principal axis of the 2*θ* mode pair, leading to modal coupling and the degradation of gyroscopic sensitivity. While both anisotropic Young’s modulus and nonideal deep trench verticality affect the frequency difference between two vibration modes, they have little contribution to deflecting the principal axis of the 2*θ* mode pair. However, the 4*θ* variations in the width of both the ring and the supporting beams cause modal coupling to occur and the degenerate 2*θ* mode pair to split in frequency. To aid the optimal design of MEMS ring-based gyroscopic sensors that has relatively high robustness to fabrication tolerance, a geometrical compensation based on the developed model is demonstrated to identify the geometries of the ring and the suspension.

## 1. Introduction

Ring-based gyroscopic sensors based on micro-electro-mechanical-system (MEMS) technology become increasingly attractive because of their high-quality factor (Q factor) and insensitivity to environmental excitation as a result of inherently symmetric structures [1,2,3]. Degenerate modes of vibratory axisymmetric ring-based structures are exploited to measure the angular rate through Coriolis coupling. When applied to external rotations about the normal axis of the ring resonator, the energy transfer occurs between the degenerate modes as a result of the Coriolis coupling effect. For a ring-based angular-rate gyroscope, one of the degenerate modes is excited with controlled amplitude and frequency, while the Coriolis force-induced vibration of the other mode is utilized to measure the angular-rate input through capacitive sensing. The ideal ring-based structures are preferred since their degenerate modes have the same resonant frequencies dictating extremely high-scale factor of the ring-based gyroscopes operating with a high Q factor [4]. The ring-based gyroscopes fabricated with isotropic materials, such as polysilicon, (111) SCS, and fused silica, are advantageous to realize mode-matching between two degenerate modes. However, the ring-based gyroscopes of isotropic materials require a more complicated and precise fabrication process than those fabricated with (100) SCS. On the other hand, the ring-based gyroscopes fabricated with (100) SCS frequently suffer from a large initial frequency split and modal coupling arising from the anisotropic Young’s modulus and the fabrication imperfections. In order to improve the dynamic performance of the imperfect ring-based gyroscopes, the post-fabrication trimming or electrostatic tuning techniques are frequently utilized for the compensation of the initial frequency split and modal coupling [5,6]. However, these techniques are sometimes not preferred due to less fidelity and efficiency. To evaluate the effects of the material anisotropy as well as the structural dimension variation, a detailed mathematical model of an imperfect ring resonator is required.

Previous works have analyzed the effects of the anisotropy of Young’ modulus on the frequency split of ring-based resonators and gyroscopes based on various numerical models utilizing the finite element method (FEM) [7,8] and analytical models [9,10], as demonstrated in Table 1. The effects of anisotropy of crystalline silicon on the vibration characteristics of ring-based resonators were accounted for in the strain energy formulation by S. McWilliam et al. [9], allowing analytical expression of the frequency split in terms of a Fourier representation of the variation in the elastic properties [7,10]. The structural imperfection of the ring-based resonators is commonly modeled as small attached masses and springs [11,12,13], which facilitate the mass trimming [14] or electrostatic tuning [6] for the reduction in frequency split. To mitigate the initial frequency split caused by the anisotropic Young’s modulus, active compensation methods based on the variation of the geometries of the ring and the suspension were proposed and proven to work effectively to some extent [7,15,16]. As can be seen, a detailed analytical model taking into account the material and structural asymmetries is still required for the development of high-performance ring-based rate or rate-integrating gyroscopes, in which circumferential uniformity and mode-matching are crucial prerequisites [17,18].

In this paper, we present a mathematical model of an imperfect ring-based gyroscope that can be utilized for the assessment of the vibration characteristics of the ring-based gyroscopes when considering the structural and material variations as illustrated in Table 1. The proposed model allows quantification of the effects of structural dimension variations and optimizing the geometrical design canceling out the effect of anisotropic Young’s modulus. Furthermore, in order to simplify the problem, we modeled both the anisotropy of Young’s modulus and the ring-based structural dimension variation in a 4*θ* periodic manner, which dominates the frequency split and modal coupling between the 2*θ* vibration modes of interest [10]. Moreover, the undesired material nonlinearity and the residue stress are not considered in this model. Based on the developed model, the effects of structural dimension variations were fully investigated and the optimized geometry that has the robustness to fabrication imperfection was proposed.

## 2. Mathematical Modeling

The ring-based Coriolis vibratory gyroscopes exploit the degenerate mode pairs of a resonator consisting of a ring, a mechanical suspension, an anchor, and a series of electrodes. A perfect ring can be regarded as a curved, continuous elastic beam whose resonance can be analyzed with the normal mode method [10]. Since the ring-based gyroscopes are usually designed and fabricated with a high aspect ratio, their out-of-plane resonances are inherently suppressed, ensuring that the simple assumption of in-plane vibration modes is still valid [19]. The in-plane resonance modes are described as *nθ* mode pairs, each pair of which is degenerate with identical natural frequencies for a perfect ring structure. In contrast, an imperfect ring will exhibit distinct but close natural frequencies for each *nθ* mode pair. The mechanical and electrostatic nonlinearity that enlarge the frequency split as the vibration amplitude increases [20,21,22] are not considered in this paper for simplicity.

**Figure 1:**
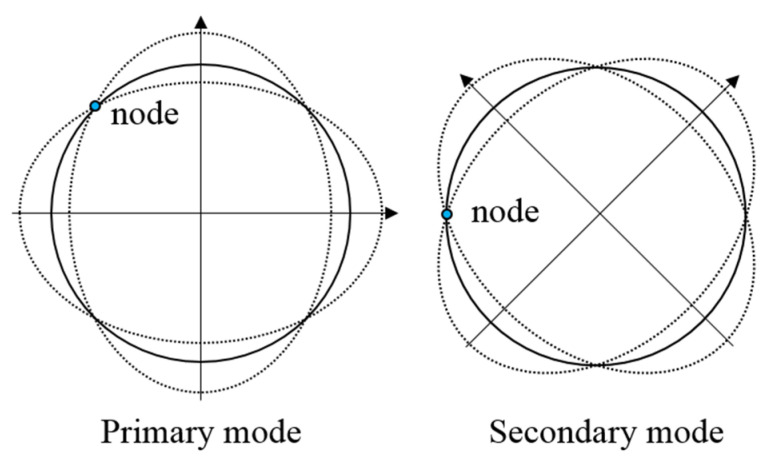
The 2*θ* mode pair of the ring-based gyroscope with elliptic mode shapes consisting of four nodes and four anti-nodes.

The resulting displacement of a ring resonator can be expressed as a weighted sum of the in-plane modes with generalized coordinates. Herein, we only consider the degenerate 2*θ* modes as shown in Figure 1, which are mostly utilized as the drive and sense modes of vibratory ring-based gyroscopes [1,3]. As a result, the displacement of the ring resonator is represented as Equation (1), where *u_r_* and *u_t_* are the radial and tangential displacement of the ring resonator, respectively, and *u_x_* and *u_y_* are the cartesian components of the displacement, respectively. Values *q*_1_ and *q*_2_ are the generalized modal coordinates.

(1) $$\begin{array}{cc} \{\begin{array}{c} u_{r}=\varphi_{r}{}_{1}q_{1}+\varphi_{r}{}_{2}q_{2}\\ u_{t}=\varphi_{t}{}_{1}q_{1}+\varphi_{t}{}_{2}q_{2} \end{array} & \{\begin{array}{c} u_{x}=\varphi_{x}{}_{1}q_{1}+\varphi_{x}{}_{2}q_{2}\\ u_{y}=\varphi_{y}{}_{1}q_{1}+\varphi_{y}{}_{2}q_{2} \end{array} \end{array}$$

The radial (*φ_r1_*, *φ_r2_*) and tangential (*φ_t1_*, *φ_t2_*) components of the 2*θ* mode shapes are expressed as:

(2) $$\left\{ \begin{array}{l} \varphi_{r}{}_{1}=\cos\left(2\theta -2\theta_{0}\right)\\ \varphi_{t}{}_{1}=-0.5\sin\left(2\theta -2\theta_{0}\right) \end{array}\right.\left\{ \begin{array}{l} \varphi_{r}{}_{2}=\sin\left(2\theta -2\theta_{0}\right)\\ \varphi_{t}{}_{2}=0.5\cos\left(2\theta -2\theta_{0}\right) \end{array}\right.$$

where *θ_0_* is the principal axis of one vibration mode of the 2*θ* mode pair, and that of the other mode is oriented 45° away from *θ_0_*; *θ* is the angular position of the ring resonator.

The cartesian components of the 2*θ* mode shapes are given by:

(3) $$\begin{array}{cc} \left[\begin{array}{c} \varphi_{xi}\\ \varphi_{yi} \end{array}\right]=\left[\begin{array}{cc} \cos\theta & -\sin\theta \\ \sin\theta & \cos\theta \end{array}\right]\left[\begin{array}{c} \varphi_{ri}\\ \varphi_{ti} \end{array}\right] & i=1,2 \end{array}$$

### 2.1. Structural Imperfection Consideration

**Figure 2:**
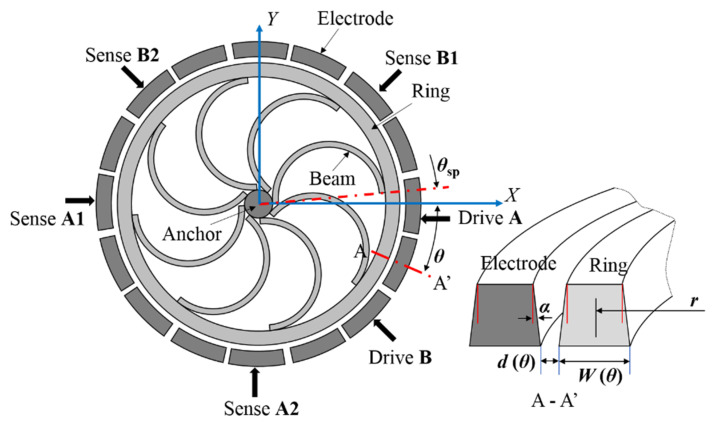
Schematic view of a ring resonator and a section of the ring and the surrounding electrode locating at *θ*.

The presence of material anisotropy and fabrication imperfection, which cannot be completely avoided, severely reduces the degeneracy and the sensitivity of ring-based gyroscopes [8]. As a result, the frequency between the drive and sense modes splits and their principal axes deflects from the predefined orientation as a result of the mass and stiffness asymmetries. This paper considers the anisotropic Young’s modulus of (100) SCS and the geometrical irregularities existed in the fabricated vibratory ring gyroscopes. As illustrated in Figure 2, the imperfect ring structure is suspended to an anchor by a mechanical suspension, composed of eight flexible semi-circular beams. The width of the ring structure varies in both circumferential and vertical directions. The sixteen surrounding electrodes are deployed for capacitive actuation, sensing, and tuning. The X and Y axes correspond to the <110> crystal directions, while the axes at 45°, 135°, 225°, and 315° are aligned to the <100> crystal direction. The ring resonator is excited by the capacitive electrode at 0° (Drive A) and the capacitive electrode at 315° (Drive B), and sensed by a differential configuration (Sense A1 and A2, Sense B1 and B2) for two vibration modes.

The Young’s modulus of the (100) SCS ring can be represented as Fourier series expansions in terms of *θ*, whose 4*θ* component is proven to dominate the frequency split between two vibration modes [10], given by:

(4) $$\begin{array}{r} E\left(\theta \right)=E_{0}+\delta E_{4}\cos\left(4\theta -4\theta_{E4}\right)\\ =E_{0}\left[1+e_{E}\cos\left(4\theta -4\theta_{E4}\right)\right] \end{array}$$

where *E*_0_, *δE*_4_ are the average and 4*θ* variation component of the Young’s modulus, respectively, *e_E_* is the variation coefficient of the Young’s modulus, and *θ_E_*_4_ is the orientation of the modulus variation with respect to the X-axis.

It is assumed that the deep trench is etched with an inclination *α* and the average ring width also varies circumferentially in a 4*θ* variation manner. The variation of the ring width and the capacitive gap between the ring and the surrounding electrodes are expressed as:

(5) $$W\left(\theta ,h\right)=W_{r}-2\alpha h+w\cos\left(4\theta -4\theta_{w}\right)$$

(6) $$d\left(\theta ,h\right)=d_{0}+2\alpha h-w\cos\left(4\theta -4\theta_{w}\right)$$

where *h* and *W_r_* are the thickness and the average radial width of the ring, respectively; *w* and *θ_w_* are the variation of the ring width and the variation orientation with respect to X-axis, respectively.

The supporting beams of the ring-based gyroscopes are frequently modeled as uniform elastic springs [10,19]. The influence of anisotropic Young’s modulus on the supporting semicircular beams is confirmed to be ineligible by modal analysis using the finite element method (FEM). As a result, we considered the irregularity of the fabricated supporting beams to be the variation of the beam width as well as the variation of the supporting location. Since the supporting beams are distributed with a pitch of 45°, they can be divided into two orthogonal groups, each of which have four beams with a pitch of 90°. One beam group has the uniform yet different beam widths with the other beam group. All supporting beams are oriented *θ_sp_* away from the corresponding electrodes. The beam width of two beam groups is shown as:

(7) $$W_{sp}=\left\{ \begin{array}{l} W_{sp0}+w_{sp}\\ W_{sp0}-w_{sp} \end{array}\right.\begin{array}{l} \mathrm{at}\\ \mathrm{at} \end{array}\begin{array}{l} \theta_{sp}+n\pi /2\\ \theta_{sp}+n\pi /2+\pi /4 \end{array}$$

where *W_sp0_* and *w_sp_* are the average beam width and the variation of beam width between two beam groups, respectively.

### 2.2. Energies of Ring-Based Gyroscope

Lagrange’s equation can be used to derive the equations of motion for the ring-based gyroscopes [10,19]. Lagrange’s equation is based on the energies of the gyroscope system including the kinetic energy of the ring, the strain energy of the ring, the potential energy of the supporting springs, the electrostatic potential energy, and the damping dissipation.

The kinetic energy of the ring is derived from:

(8) $$T=\frac{1}{2}\int_{V}\rho \left[{\dot{u}}_{x}^{2}+{\dot{u}}_{y}^{2}+2\Omega_{z}\left(u_{x}{\dot{u}}_{y}-u_{y}{\dot{u}}_{x}\right)\right]dV$$

where *ρ* is the density of the ring material, *V* is the volume of the ring, and Ω*_Z_* is the angular rate about the axis of the ring.

The kinetic energy of the ring is obtained by substituting Equation (1) into (8):

(9) $$T=\frac{1}{2}M_{11}{\dot{q}}_{1}^{2}+\frac{1}{2}M_{22}{\dot{q}}_{2}^{2}+M_{12}{\dot{q}}_{1}{\dot{q}}_{2}+\Omega_{z}\gamma \left(q_{1}{\dot{q}}_{2}-q_{2}{\dot{q}}_{1}\right)$$

where *A* is the area of the cross-section of the ring, and,

(10) $$\begin{array}{cc} M_{ij}=\int_{V}\rho \left(\varphi_{xi}\varphi_{xj}+\varphi_{yi}\varphi_{yj}\right)\cdot Ad\theta & \left(i,j=1,2\right) \end{array}$$

(11) $$\gamma =\int_{V}\rho \left(\varphi_{x1}\varphi_{y2}-\varphi_{x2}\varphi_{y1}\right)\cdot Ad\theta$$

Taking into account of the asymmetries of the ring, the mass *M_ij_* in Equation (10) becomes:

(12) $$M_{11}=\frac{5}{4}\pi \rho W_{r}^{'}H_{r}R_{r}\left(1+\frac{3}{10}e_{w}\cos\left(4\phi_{w}\right)\right)$$

(13) $$M_{22}=\frac{5}{4}\pi \rho W_{r}^{'}H_{r}R_{r}\left(1-\frac{3}{10}e_{w}\cos\left(4\phi_{w}\right)\right)$$

The modal coupling in terms of inertia can be neglected since the variation of the ring width is negligibly small when compared to the ring width itself. Therefore, *M*_12_ and *M*_21_ are considered to be zero in the following discussions. Similarly, the in-plane displacement of the ring is negligibly small when compared to the radial thickness. As a result, the ring structure can be considered to be inextensible and linear, ensuring that the bending strain energy is dominant [19].

The change in curvature of each ring element due to bending can be expressed as:

(14) $$\frac{1}{r+\Delta r}-\frac{1}{r}=\frac{\left({x^{\prime }}_{r}+{u^{\prime }}_{x}\right)\left({y^{\prime \prime }}_{r}+{u^{\prime \prime }}_{y}\right)}{{\left({x^{\prime }}_{r}{}^{2}+{y^{\prime }}_{r}{}^{2}\right)}^{3/2}}-\frac{\left({y^{\prime }}_{r}+{u^{\prime }}_{y}\right)\left({x^{\prime \prime }}_{r}+{u^{\prime \prime }}_{x}\right)}{{\left({x^{\prime }}_{r}{}^{2}+{y^{\prime }}_{r}{}^{2}\right)}^{3/2}}-\frac{\left({x^{\prime }}_{r}y^{\prime \prime }{}_{r}-{y^{\prime }}_{r}{x^{\prime \prime }}_{r}\right)}{{\left({x^{\prime }}_{r}{}^{2}+{y^{\prime }}_{r}{}^{2}\right)}^{3/2}}$$

As a result, the bending strain energy of the entire ring structure is obtained by integrating the bending energy over the ring elements, given by:

(15) $$U_{r}=\int_{V}EI{\left[\frac{1}{r+\Delta r}-\frac{1}{r}\right]}^{2}ds$$

where *E* is the Young’s modulus and *I* = *w*^3^*t* / 12 is the second moment of area. Replacing the change in curvature of each ring element in Equation (14), the bending energy can be shown as:

(16) $$U_{r}=\frac{1}{2}Kr_{11}q_{1}^{2}+\frac{1}{2}Kr_{22}q_{2}^{2}+Kr_{12}q_{1}q_{2}$$

where *Kr_ij_* represents the mechanical stiffness of the ring structure, given by:

(17) Krij=∫02πEI(x′rφ″yi−y′rφ″xi+y″rφ′xi−x″rφ′yi)⋅(x′rφ″yj−y′rφ″xj+y″rφ′xj−x″rφ′yj)/(x′r2+y′r2)52dθ

For simplicity, each semicircular supporting beam is modeled by incorporating one radial and one tangential spring to the ring structure. Based on static equilibrium analysis, the radial stiffness (*Kb_r_*) and the tangential stiffness (*Kb_t_*) of the semicircular beam are given by [10]:

(18) $$\frac{1}{Kb_{r}}=\frac{r_{sp}^{3}}{E_{0}I_{sp}}\left(\frac{\pi }{2}-\frac{4}{\pi }\right)$$

(19) $$\frac{1}{Kb_{t}}=\frac{r_{sp}^{3}}{E_{0}I_{sp}}\left(\frac{\pi }{2}\right)$$

The potential energy of the support springs can be derived by summing the potential energies of eight beams, given by:

(20) $$U_{sp}=\sum_{8}\left(\frac{1}{2}Kb_{r}u_{r}{}^{2}+\frac{1}{2}Kb_{t}u_{t}{}^{2}\right)$$

Replacing the displacement of the ring at the positions where the supporting springs are attached in Equation (1), it can be shown as:

(21) $$U_{sp}=\frac{1}{2}K_{sp11}q_{1}^{2}+\frac{1}{2}K_{sp22}q_{2}^{2}+K_{sp12}q_{1}q_{2}$$

where *K_spij_* (*i*, *j* =1, 2) represents the effective stiffness of the supporting springs by the expression:

(22) $$\begin{array}{cc} K_{spij}=\sum_{8}\left(Kb_{r}\varphi_{ri}\varphi_{rj}+Kb_{t}\varphi_{ti}\varphi_{tj}\right) & \left(i,j=1,2\right) \end{array}$$

The capacitive electrodes for the drive and sense of the ring gyroscope are shown in Figure 2. Considering the imperfections of the ring and the capacitive gap, the capacitors cannot be simply approximated as parallel plate capacitors. For a non-parallel plate capacitor, the capacitance for an electrode pair at *θ_n_* is given by:

(23) $$C_{n}≅\int_{\theta_{n}-\frac{\Delta \theta_{n}}{2}}^{\theta_{n}+\frac{\Delta \theta_{n}}{2}}\int_{0}^{H_{r}}\frac{\varepsilon R_{r}dhd\theta }{d_{0}+2\alpha h-w\cos\left(4\theta -4\theta_{w}\right)+\Delta d}$$

where Δ*d* represents the radial displacement of the ring element.

By integrating Equation (23) along *h*, the capacitance is expressed as:

(24) $$C_{n}≅\int_{\theta_{n}-\frac{\Delta \theta_{n}}{2}}^{\theta_{n}+\frac{\Delta \theta_{n}}{2}}\frac{\varepsilon R_{r}}{2\alpha }\ln\left(1+\frac{2\alpha H_{r}}{d_{0}+\Delta d^{\prime }}\right)d\theta$$

To facilitate the development of linear analytical expression that takes into account of all the structural imperfections, the integrated term in Equation (24) is approximated by the linear terms of its Taylor series expansion:

(25) $$C_{n}≅C_{sn}+q_{1}f1_{n}+q_{2}f2_{n}+q_{1}^{2}g1_{n}+q_{2}^{2}g2_{n}+2q_{1}q_{2}h_{n}$$

The full expression of each component in Equation (25) is shown in Appendix A. The total electrostatic potential energies arising from the drive and sense electrodes can be derived as:

(26) $$U_{e}=-\sum_{1}\frac{1}{2}C_{dn}{\left(V_{d}-V_{p}\right)}^{2}-\sum_{2}\frac{1}{2}C_{sn}{\left(V_{b}-V_{p}\right)}^{2}$$

Performing the integration with respect to *θ* by using Equation (26), the expression of the electrostatic energies for both the drive and sense electrodes can be determined.

The damping dissipation of the ring gyroscope occurs from the air damping, the thermoelastic damping, the support loss, and so on [23]. In vacuum condition, the dominant damping factor comes from the thermoelastic damping that can be approximated via Rayleigh’s dissipation function [24]. For simplicity, only the linear damping coefficient is considered in this paper and calculated based on the measured Q factor values. The coupling terms in the damping matrix are assumed to be zero.

### 2.3. Governing Equations of Motion

The governing equations of motion are derived by substituting the kinetic and potential energy expressions into the Lagrange’s equation and performing the required differentiation. *T* represents the kinetic energy of the ring, *U* represents the total potential energies including the strain potential of the ring, the potential energy of the supporting springs and the electrostatic potential energies of the drive and sense electrodes, and *W* represents the dissipation arising from the proportional damping:

(27) $$\frac{d}{dt}\frac{\partial T}{\partial {\dot{q}}_{j}}-\frac{\partial T}{\partial q_{j}}+\frac{\partial U}{\partial {\dot{q}}_{j}}=\frac{\partial W}{\partial {\dot{q}}_{j}}(j=1,2)$$

(28) $$\begin{array}{c} \overset{mass}{\left[\begin{array}{cc} M_{11} & M_{12}\\ M_{21} & M_{22} \end{array}\right]}\left\{\begin{array}{c} {\ddot{q}}_{1}\\ {\ddot{q}}_{2} \end{array}\right\}+\overset{damping}{\left[\begin{array}{cc} c_{11} & c_{12}\\ c_{21} & c_{22} \end{array}\right]}\left\{\begin{array}{c} {\dot{q}}_{1}\\ {\dot{q}}_{2} \end{array}\right\}+\overset{\begin{array}{cc} Coriolis & coupling \end{array}}{\left[\begin{array}{cc} 0 & -2\gamma \Omega_{z}\\ 2\gamma \Omega_{z} & 0 \end{array}\right]}\left\{\begin{array}{c} {\dot{q}}_{1}\\ {\dot{q}}_{2} \end{array}\right\}+\overset{\begin{array}{cc} ring & structure \end{array}}{\left[\begin{array}{cc} Kr_{11} & Kr_{12}\\ Kr_{21} & Kr_{22} \end{array}\right]}\left\{\begin{array}{c} q_{1}\\ q_{2} \end{array}\right\}+\\ \overset{spring}{\left[\begin{array}{cc} K_{sp11} & K_{sp12}\\ K_{sp21} & K_{sp22} \end{array}\right]}\left\{\begin{array}{c} q_{1}\\ q_{2} \end{array}\right\}+\overset{\begin{array}{cc} electrostatic & stiffness \end{array}}{\left[\begin{array}{cc} Ke_{11} & Ke_{12}\\ Ke_{21} & Ke_{22} \end{array}\right]}\left\{\begin{array}{c} q_{1}\\ q_{2} \end{array}\right\}=\overset{\begin{array}{cc} drive & force \end{array}}{\left\{\begin{array}{c} f_{1}\\ f_{2} \end{array}\right\}} \end{array}$$

where *Ke_ij_* represents the negative stiffness arising from the electrostatic spring softening effects.

The full expressions of mass, damping, stiffness, Coriolis coupling, and force terms of the vibratory ring gyroscope are shown in the Appendix A.

## 3. Modal Analysis

To determine the influence of the above-mentioned asymmetries on the response of the 2*θ* generalized coordinates, the differential Equation (27) is solved analytically using MATLAB (2017b, MathWorks, Natick, MA, USA). The physical properties of the vibratory ring gyroscope and the electronic parameters are shown in Table 2.

The flexural 2*θ* vibrations can be described in terms of two normal vibration modes. The mode coupling in terms of stiffness, arising from the structural imperfection, contributes to the rotations of the principal axes of vibration modes. As a result, the angle of the principal axis (*θ*_0_) with respect to the 0° pickoff electrode can be determined by assigning the coupling stiffness terms to be zero [25,26]:

(29) $$K_{r12}+K_{sp12}=K_{r21}+K_{sp21}=0$$

where the electrostatic coupling stiffness term is negligibly small when compared to that of the elastic stiffness.

By solving Equation (29), the principal axis angle with respect to the 0° pickoff electrode is given by:

(30) $$\theta_{0}=\frac{1}{4}\arctan\left(\frac{e_{r1}K_{r0}+e_{sp1}\left(4Kb_{r}-Kb_{t}\right)}{e_{r2}K_{r0}+e_{sp2}\left(4Kb_{r}-Kb_{t}\right)}\right)$$

where the full expression of *e_r_*_1_, *e_r_*_2_, *e_sp_*_1_, and *e_sp_*_2_ is shown in the Appendix A.

In addition to modal coupling, the frequencies of two vibration modes split in response to the structural asymmetry as well as the material anisotropy. The electrostatic excitation imposed on the electrodes produces an effect of electrostatic spring softening, which is commonly utilized to eliminate the frequency split of the overall system. Therefore, the electrostatic negative stiffness is also included to derive the frequency split between 2*θ* resonant modes, shown as:

(31) $$\Delta f=\frac{1}{2\pi }(\sqrt{\frac{K_{r11}+K_{sp11}+K_{e11}}{M_{11}}}-\sqrt{\frac{K_{r22}+K_{sp22}+K_{e22}}{M_{22}}})$$

To verify the validation of the developed analytical model, FEM simulations using COMSOL Multiphysics (5.6, COMSOL Inc, Burlington, MA, USA) are also used to verify the analytical results. To verify the reported geometrical compensation methods, modal analyses using FEM are performed for imperfect rings in which the ring width variation is derived based on the anisotropic Young’s modulus shown in Equation (4).

## 4. Results and Discussion

### 4.1. Frequency Split

The influence of anisotropic Young’s modulus on the frequency split of the ring gyroscope is first investigated by solving Equation (31), assuming a perfect ring and suspension with uniform structural dimension. The estimated frequency split between two vibration modes is around 593 Hz for the ring-based gyroscope, which is in good agreement with the measured frequency split of about 625 Hz (*σ* = 18 Hz). The little discrepancy can be attributed to fabrication imperfection. For comparison, the frequency split arising from the asymmetry of Young’s modulus is estimated by a reported analytical expression [9] as:

(32) $$\Delta \omega =\frac{1}{2}e_{4}\omega_{0}$$

where *ω*_0_ is the natural frequency of the ring gyroscopes, *E_0_* is the averaged Young’s modulus, and *e*_4_ is the 4*θ* variation component.

As shown in Table 3, the estimated frequency split of the ring gyroscope by using Equation (31) and that predicted by FEM exhibit relatively higher values, probably due to the negligence of the contribution from the electrostatic forces. As expected, the principal axis of the 2*θ* mode pair is not affected by the anisotropic Young’s modulus. It can be proven that the anisotropy of Young’s modulus plays a dominant role in the frequency split between two vibration modes of the ring gyroscope, which can be accurately predicted by the proposed mathematical model.

Considering the nonvertical deep trench of the fabricated ring gyroscope, the natural frequencies of two vibration modes reduce as the trench angle variation increases as expected. Due to the assumption of uniform inclination of the capacitive gap, the principal axis of the 2*θ* mode pair remains unaltered with respect to the 0° pickoff electrode. The frequency split between two vibration modes reduces by about 79 Hz as the inclination angle decreases 1° from 90°. As a result of the improvement of the fabrication technology, the trench verticality of the ring gyroscope can be guaranteed, ensuring a relatively negligible influence on the frequency split compared to the effect of anisotropic Young’s modulus.

**Figure 3:**
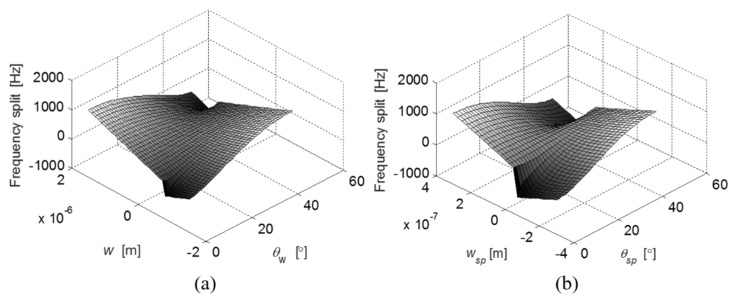
The influence of the width and orientation of the ring (**a**) and the supporting beams (**b**) on the frequency split.

By varying the structural dimensions of the ring and suspension structures individually, the effects on the frequency split are quantified based on Equation (30). Figure 3a,b illustrate the frequency split arising from the variation of the ring geometry and the variation of the suspension geometry, respectively. The 4*θ* periodic manner of the ring/beam width variation determines a periodic frequency split when either *θ_w_* or *θ_sp_* changes every 90°. The maximum frequency split occurs when the width ring/beam variation (*w* or *w_sp_*) in the <110> crystal direction is larger than that in the <100> crystal direction as expected. The frequency split can be reduced to zero when either the ring structure or the supporting beams are assigned with specific widths and orientations.

**Figure 4:**
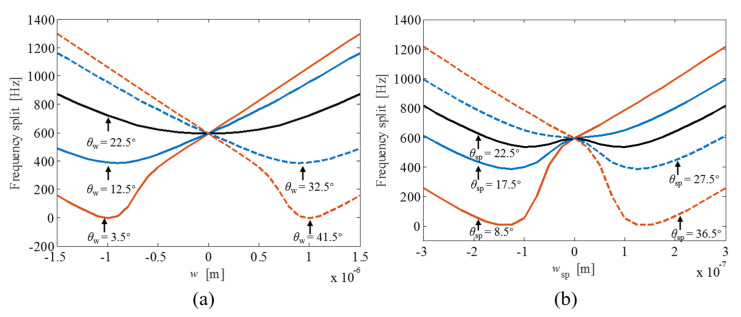
The influence of the width variation of both the ring (**a**) and the supporting beams (**b**) on the frequency split.

The effects of the ring/beam width variation of the ring-based gyroscope on the frequency split are shown in Figure 4. When the variation orientation (*θ_w_*) of the ring is less than 3.5° or more than 41.5°, the frequencies of two vibration modes can be exactly matched with specific width variation of the ring structure. However, the frequency split in response to the width variation of the ring structure does not exhibit a mode-matched state without frequency split when the variation orientation (*θ_w_*) of the ring ranges from 4° to 41°. The mode-matching state is similarly not exhibited for the case of the supporting beams with their variation orientation (*θ_sp_*) assigned between 9° and 36°. When both θ_w_ and *θ_sp_* are located at 22.5°, the frequency split has a symmetric and relatively flat response to the width variation of both the ring structure and the supporting beams. This indicates that the frequencies of both vibration modes are similarly affected by the ring/beam width variation at this specific variation orientation. When *θ_w_* and *θ_sp_* deflect from 22.5°, the frequency split as a function of the width variation *w* or *w_sp_* exhibits a “spoon”-shaped tendency. The bottom of the “spoon” intersects with the mode-matched line when *θ_w_* ≤ 3.5° or *θ_sp_* ≥ 41.5°. In addition, the variation of the beam width has a relatively larger contribution to the frequency split than the variation of the ring width because of the distributed property of the ring structure.

### 4.2. Modal Coupling

**Figure 5:**
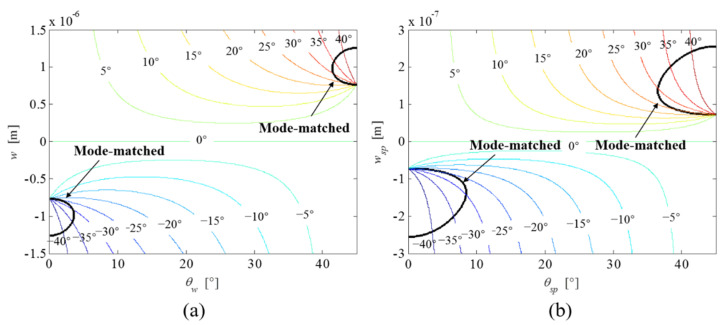
The influence of width variation of both the ring (**a**) and the supporting beams (**b**) on the principal axis of the 2*θ* mode pair, which is represented by contour maps. The black curved lines indicate the mode-matched solutions.

The principal axis of the 2*θ* mode pair, at the same time, rotates either clockwise or anti-clockwise in response to the structural variation of the ring structure and supporting beams. As shown in Figure 5, except for a perfect ring gyroscope, the principal axis of the 2*θ* mode pair rotates in a clockwise or anti-clockwise direction depending on the width variation *w* and *w_sp_*. When either *w* and *w_sp_* is larger than zero, the principal axis of the 2*θ* mode pair deflects in an anti-clockwise direction, and vice versa. When the width variations *w* and *w_sp_* are larger than 1.5 and 0.3 μm, respectively, the principal axis of the 2*θ* mode pair is dominated by their variation orientations (*θ_w_* and *θ_sp_*). As the black curved line indicated in Figure 5 two vibration modes are matched with specific variations of the width and orientation. For example, the mode-matched state occurs when *w* ranges from −0.75 to −1.25 μm, and *θ_w_* ranges from 0° to 3.5°. Accordingly, the principal axis of the 2*θ* mode pair with respect to the 0° pickoff electrode varies from 0° to ±45°. Modal coupling arises from the deflection of the principal axis of the 2*θ* mode pair with respect to predefined electrode configuration. The principal axis of the mode-matched 2*θ* mode pair becomes least insensitive to the width variation when *θ_w_* and *θ_sp_* are 3.5° and 8.5°, respectively.

### 4.3. Geometrical Compensation

It requires precise geometrical compensation of the ring structure and the supporting beams to eliminate the frequency split between two vibration modes arising from material anisotropy. However, fabrication imperfection cannot be avoided completely even with precise and sophisticated processes. To reduce the effects of fabrication tolerance, it is required that the geometrical design of the ring gyroscope is insensitive and robust to the geometrical variation, ensuring the fabricated sensors having a minimum frequency split with little variation. As can be derived from the developed model, the vibratory ring gyroscope with the structural dimension variation of the ring structure, such as *θ_w_* = 3.5° and *w* = −1.0 μm, has no frequency split between two vibration modes in theory, which varies less than 13 Hz, given a fabrication tolerance of *w* within ±0.1 μm. At the same time, the principal axis of the 2*θ* mode pair is about −32.5 ± 3.5° with the same fabrication tolerance. On the other hand, the compensation of frequency split through the structural dimension variation of the supporting beams is much less robust to the fabrication tolerance and results into much larger deflection of the principal axis of the 2*θ* mode pair. Two other reported geometrical compensation methods [15,16] for the ring-based gyroscopes based on the compensation method through the variation of the ring width were evaluated, by which the estimated frequency splits are more than 250 Hz and 400 Hz, respectively, as illustrated in Table 4. Therefore, the proposed geometrical compensation based on the developed model is advantageous due to its prediction accuracy and robustness to fabrication tolerance.

## 5. Conclusions

A mathematical model was presented for a ring-based gyroscope with considering the structural dimension variation and material anisotropy. The developed model is simplified with assuming linear material, constant damping, and residual stress-free properties. Furthermore, investigation about the effects on the vibration response in terms of the frequency split and modal coupling was performed. The predominant frequency split between two 2*θ* modes caused by the anisotropy of Young’s modulus was predicted using the developed model, in good agreement with measurements. Moreover, the resonance asymmetry of the ring-based gyroscope was parametrically studied through the structural dimension variations of both the ring structure and the supporting beams independently, arising from the fabrication imperfection or active design. The verticality variation of the deep trench as a result of fabrication imperfection slightly affects the frequency split of two vibration modes, exhibiting little influence on the principal axis of the 2*θ* mode pair. Mode-matching can be realized for the ring-based gyroscope by varying the lateral dimension of the ring structure, except the orientation of the 4*θ* variation (*θ_w_*) locating between 4° and 41°. This frequency split-canceling effect is similarly exhibited for the structural dimension variation of the supporting beams, which requires more precise control of the beam width. At the same time, the lateral dimension variations of both the ring and the supporting beams induce modal coupling in terms of the rotation of the principal axis with respect to the predefined electrodes. Based on the above analysis, the optimal structural dimension of the vibratory ring gyroscope that guarantees a small range of frequency split and the deflection of the principal axis are obtained, necessitating robust fabrication and post-fabrication electrostatic tuning.

## Figures and Tables

**Table 1:** Models of ring-based gyroscopes.

Model	Material Anisotropy	Mass Asymmetry	Non-Ideal Ring	Non-Ideal Beam	Non-Ideal Trench	Reference
FEM	Yes	Yes	Yes	Yes	No	[7]
FEM	Yes	Yes	Yes	No	No	[8]
Analytical	Yes	No	No	No	No	[9]
Analytical	Yes	Yes	No	No	No	[10]
Analytical	Yes	Yes	Yes	Yes	Yes	This work

**Table 2:** Properties of ring-based gyroscopes.

Parameter	Value	Description
*E*_0_ (GPa)	150	Average Young’s modulus of (100) SCS
*ρ* (kg/m3)	2330	Bulk density
*R_r_* (mm)	1	Ring radius
*W_r_* (μm)	11	Ring width
*H_r_* (μm)	60	Ring thickness
Δ*θ* (°)	18	Electrode radian
*r_sp_* (μm)	472.5	Beam radius
*W_sp_* (μm)	5	Beam width
*d_0_* (μm)	4	Electrode and undeformed ring gap
*C_inp_* (pF)	1	Feedback reference capacitance in CV circuit
*V_dn_* (V)	2.5	Drive voltage
*V_p_* (V)	2.5	Polarization voltage
*V_b_* (V)	0	Bias voltage at the pick-off

**Table 3:** Frequency split considering anisotropic Young’s modulus.

Method	Frequency Split (Hz)	Description
Analytical	844	Equation (32)
Analytical	593	Equation (31)
FEM	799	COMSOL
Experimental	625 (σ =18 Hz)	nine as fabricated samples

**Table 4:** Geometrical compensation of ring-based gyroscopes.

Structural Dimension	Method	Frequency Split [Hz]	Description
Ring Width	$W(\theta )=W_{\left(100\right)}{\left(\frac{E_{100}}{E(\theta )}\right)}^{1/3}$	414 (FEM)	[16]
	$W(\theta )=W_{\left(100\right)}{\left(\frac{E_{100}}{E(\theta )}\right)}^{1/2}$	255 (FEM)	[15]
	*W*(*θ*, *h*) = *W_r_* − 2*αh* + *w* cos(4*θ* − 4*θ*_w_)	<10 (Analytical) ^1^	In this paper

^1^ Given a fabrication tolerance of *w* within ±0.1 μm.

## Appendix A

The capacitance of an electrode pair at *θ_n_* is given by:

(A1) $$\begin{array}{l} C_{n}≅\frac{\varepsilon H_{r}R_{r}}{d_{0}}\left(1-e_{\alpha }\right)\int_{\theta_{n}-\frac{\Delta \theta_{n}}{2}}^{\theta_{n}+\frac{\Delta \theta_{n}}{2}}d\theta -\\ \;\frac{\varepsilon H_{r}R_{r}}{d_{0}^{2}}\left(1-2e_{\alpha }\right)\int_{\theta_{n}-\frac{\Delta \theta_{n}}{2}}^{\theta_{n}+\frac{\Delta \theta_{n}}{2}}\Delta d^{\prime }d\theta +\frac{\varepsilon H_{r}R_{r}}{d_{0}^{3}}\left(1-3e_{\alpha }\right)\int_{\theta_{n}-\frac{\Delta \theta_{n}}{2}}^{\theta_{n}+\frac{\Delta \theta_{n}}{2}}\Delta {d^{\prime }}^{2}d\theta \\ \;≅C_{s}+q_{1}f1_{n}+q_{2}f2_{n}+q_{1}^{2}g1_{n}+q_{2}^{2}g2_{n}+2q_{1}q_{2}h_{n} \end{array}$$

The full expression of each component in Equation (25) is shown as:

(A2) $$\begin{array}{l} C_{s}=\left(1-e_{\alpha }\right)\frac{\varepsilon H_{r}R_{r}}{d_{0}}\Delta \theta_{n}\left(1+\frac{1}{2}e_{d}^{2}\Delta \theta_{n}\right)+\left\{\begin{array}{l} \left(1-e_{\alpha }\right)\frac{\varepsilon H_{r}R_{r}}{d_{0}}\Delta \theta_{n}\\ \cdot \frac{1}{2}e_{d}\left[\begin{array}{l} \cos\left(4\theta -4\theta_{w}\right)\sin\left(2\Delta \theta_{n}\right)+\\ \frac{1}{4}e_{d}\cos\left(8\theta -8\theta_{w}\right)\sin\left(4\Delta \theta_{n}\right) \end{array}\right] \end{array}\right\}\\ f1_{n}=a\cos\left(2\theta -2\theta_{0}\right)\cdot 2e_{d}\cos(4\phi_{w})\cdot \left[\begin{array}{l} 1-2\sin^{2}\left(2\theta -2\theta_{0}\right)\cos^{2}\left(\Delta \theta_{n}\right)-\\ \frac{2}{3}\cos^{2}\left(2\theta -2\theta_{0}\right)\sin^{2}\left(\Delta \theta_{n}\right) \end{array}\right]+\\ a\sin\left(2\theta -2\theta_{0}\right)\cdot 4e_{d}\sin(4\phi_{w})\cdot \left[\begin{array}{l} \cos^{2}\left(2\theta -2\theta_{0}\right)\cos^{2}\left(\Delta \theta_{n}\right)+\\ \frac{1}{3}\sin^{2}\left(2\theta -2\theta_{0}\right)\sin^{2}\left(\Delta \theta_{n}\right) \end{array}\right]+a\cos\left(2\theta -2\theta_{0}\right)\\ f2_{n}=-a\sin\left(2\theta -2\theta_{0}\right)\cdot 2e_{d}\cos(4\phi_{w})\cdot \left[\begin{array}{l} 1-2\cos^{2}\left(2\theta -2\theta_{0}\right)\cos^{2}\left(\Delta \theta_{n}\right)-\\ \frac{2}{3}\sin^{2}\left(2\theta -2\theta_{0}\right)\sin^{2}\left(\Delta \theta_{n}\right) \end{array}\right]+\\ a\cos\left(2\theta -2\theta_{0}\right)\cdot 4e_{d}\sin(4\phi_{w})\cdot \left[\begin{array}{l} \sin^{2}\left(2\theta -2\theta_{0}\right)\cos^{2}\left(\Delta \theta_{n}\right)+\\ \frac{1}{3}\cos^{2}\left(2\theta -2\theta_{0}\right)\sin^{2}\left(\Delta \theta_{n}\right) \end{array}\right]+a\sin\left(2\theta -2\theta_{0}\right)\\ g1_{n}=\left(1-3e_{\alpha }\right)\frac{\varepsilon H_{r}R_{r}}{d_{0}^{3}}\cdot \left[\frac{\Delta \theta_{n}}{2}+\frac{1}{4}\cos\left(4\theta -4\theta_{0}\right)\sin\left(2\Delta \theta_{n}\right)\right]\\ g2_{n}=\left(1-3e_{\alpha }\right)\frac{\varepsilon H_{r}R_{r}}{d_{0}^{3}}\cdot \left[\frac{\Delta \theta_{n}}{2}-\frac{1}{4}\cos\left(4\theta -4\theta_{0}\right)\sin\left(2\Delta \theta_{n}\right)\right]\\ h_{n}=\left(1-3e_{\alpha }\right)\frac{\varepsilon H_{r}R_{r}}{d_{0}^{3}}\frac{1}{4}\sin\left(4\theta -4\theta_{0}\right)\sin\left(2\Delta \theta_{n}\right) \end{array}$$

where:

(A3) $$a=\left(1-2e_{\alpha }\right)\frac{\varepsilon H_{r}R_{r}}{d_{0}^{2}}\sin\left(\Delta \theta_{n}\right)$$

The off-diagonal and cross-diagonal terms in the mass matrix are shown as follows. The term *M*_12_ and *M*_21_ representing in-pair modal coupling in terms of inertia is negligible when the width variation is small when compared to the width.

(A4) $$\left\{ \begin{array}{l} M_{11}=\frac{5}{4}\pi \rho W_{r}^{'}H_{r}R_{r}\left(1+\frac{3}{10}e_{w}\cos\left(4\phi_{w}\right)\right)\\ M_{22}=\frac{5}{4}\pi \rho W_{r}^{'}H_{r}R_{r}\left(1-\frac{3}{10}e_{w}\cos\left(4\phi_{w}\right)\right)\\ M_{12}=M_{21}=-\frac{3}{8}e_{w}\sin\left(4\phi_{w}\right)\pi \rho W_{r}^{'}H_{r}R_{r} \end{array}\right.$$

The term *γ* arising from Coriolis coupling when the ring rotates about the polar axis is:

(A5) $$\gamma =\pi \rho W_{r}^{'}H_{r}R_{r}$$

where:

(A6) $${W^{\prime }}_{r}=W_{r}-\alpha H_{r}$$

The stiffness coefficients of the ring structure are:

(A7) $$\left\{ \begin{array}{l} Kr_{11}=K_{r0}\left\{\begin{array}{l} e_{1}+e_{1}e_{E}\sin\left(4\phi_{Ew}\right)\sin\left(4\phi_{w}\right)+\frac{1}{2}e_{2}\left[e_{E}\cos\left(4\phi_{Ew}\right)+\cos\left(4\phi_{w}\right)\right]+\\ \frac{1}{2}e_{3}e_{E}\cos\left(4\phi_{Ew}+4\phi_{w}\right) \end{array}\right\}\\ Kr_{22}=K_{r0}\left\{\begin{array}{l} e_{1}-e_{1}e_{E}\sin\left(4\phi_{Ew}\right)\sin\left(4\phi_{w}\right)+\frac{1}{2}e_{2}\left[e_{E}\cos\left(4\phi_{Ew}\right)-\cos\left(4\phi_{w}\right)\right]-\\ \frac{1}{2}e_{3}e_{E}\cos\left(4\phi_{Ew}+4\phi_{w}\right) \end{array}\right\}\\ Kr_{12}=Kr_{21}=K_{r0}\left[-e_{1}e_{E}\sin\left(4\phi_{Ew}\right)\cos\left(4\phi_{w}\right)+\frac{1}{2}e_{2}\sin\left(4\phi_{w}\right)+\frac{1}{2}e_{3}e_{E}\sin\left(4\phi_{Ew}+4\phi_{w}\right)\right] \end{array}\right.$$

where:

(A8) $$\left\{ \begin{array}{l} e_{w}=w/W_{r}^{'}\\ e_{t}=W_{r}/W_{r}^{'}\\ e_{wsp}=w_{sp}/W_{sp}\\ \phi_{Ew}=\theta_{w}-\theta_{E4}\\ \phi_{sp}=\theta_{sp}-\theta_{0}\\ \phi_{w}=\theta_{w}-\theta_{0}\\ e_{1}=e_{t}^{2}-2e_{t}+2+e_{w}^{2}\\ e_{2}=\left(e_{1}+1-0.25e_{w}^{2}\right)e_{w}\\ e_{3}=e_{1}+0.5e_{w}^{2}\\ e_{4}=e_{wsp}\left(e_{wsp}^{2}+3\right) \end{array}\right.$$

The stiffness coefficients of the support springs are:

(A9) $$\left\{ \begin{array}{l} K_{sp11}=\left(1+3e_{wsp}^{2}\right)\left(4K_{HA0}+K_{VA0}\right)+e_{4}\cos\left(4\phi_{sp}\right)\left(4K_{HA0}-K_{VA0}\right)\\ K_{sp11}=\left(1+3e_{wsp}^{2}\right)\left(4K_{HA0}+K_{VA0}\right)-e_{4}\cos\left(4\phi_{sp}\right)\left(4K_{HA0}-K_{VA0}\right)\\ K_{sp12}=K_{sp21}=e_{4}\sin\left(4\phi_{sp}\right)\left(4K_{HA0}-K_{VA0}\right) \end{array}\right.$$

The linear electrostatic stiffness coefficients are:

(A10) $$\left\{ \begin{array}{l} Ke_{11}=-2cV_{p}^{2}-4cV_{s}^{2}+4ab\left[\begin{array}{l} {\left\{\begin{array}{l} \cos\left(2\theta_{0}\right)\left[1+2e_{d}\cos\left(4\phi_{w}\right)\left(1-2\beta \right)\right]-\\ \sin\left(2\theta_{0}\right)\left[4e_{d}\sin\left(4\phi_{w}\right)\mu \right] \end{array}\right\}}^{2}+\\ {\left\{\begin{array}{l} \sin\left(2\theta_{0}\right)\left[1+2e_{d}\cos\left(4\phi_{w}\right)\left(1-2\mu \right)\right]\\ +\cos\left(2\theta_{0}\right)\left[4e_{d}\sin\left(4\phi_{w}\right)\beta \right] \end{array}\right\}}^{2} \end{array}\right]V_{s}^{2}\\ Ke_{22}=-2cV_{p}^{2}-4cV_{s}^{2}+4ab\left[\begin{array}{l} {\left\{\begin{array}{l} \sin\left(2\theta_{0}\right)\left[1-2e_{d}\cos\left(4\phi_{w}\right)\left(1-2\mu \right)\right]-\\ \cos\left(2\theta_{0}\right)\left[4e_{d}\sin\left(4\phi_{w}\right)\beta \right] \end{array}\right\}}^{2}+\\ {\left\{\begin{array}{l} \cos\left(2\theta_{0}\right)\left[1-2e_{d}\cos\left(4\phi_{w}\right)\left(1-2\beta \right)\right]+\\ \sin\left(2\theta_{0}\right)\left[4e_{d}\sin\left(4\phi_{w}\right)\mu \right] \end{array}\right\}}^{2} \end{array}\right]V_{s}^{2}\\ Ke_{12}=Ke_{21}=-2ab\left\{\begin{array}{l} \left\{\cos\left(2\theta_{0}\right)\left[1+2e_{d}\cos\left(4\phi_{w}\right)\left(1-2\beta \right)\right]-\sin\left(2\theta_{0}\right)\left[4e_{d}\sin\left(4\phi_{w}\right)\mu \right]\right\}\cdot \\ \left\{\sin\left(2\theta_{0}\right)\left[1-2e_{d}\cos\left(4\phi_{w}\right)\left(1-2\mu \right)\right]-\cos\left(2\theta_{0}\right)\left[4e_{d}\sin\left(4\phi_{w}\right)\beta \right]\right\}-\\ \left\{\sin\left(2\theta_{0}\right)\left[1+2e_{d}\cos\left(4\phi_{w}\right)\left(1-2\mu \right)\right]+\cos\left(2\theta_{0}\right)\left[4e_{d}\sin\left(4\phi_{w}\right)\beta \right]\right\}\cdot \\ \left\{\cos\left(2\theta_{0}\right)\left[1-2e_{d}\cos\left(4\phi_{w}\right)\left(1-2\beta \right)\right]-\sin\left(2\theta_{0}\right)\left[4e_{d}\sin\left(4\phi_{w}\right)\mu \right]\right\} \end{array}\right\}V_{s}^{2} \end{array}\right.$$

where:

(A11) $$\left\{ \begin{array}{l} a=\left(1-2e_{\alpha }\right)\frac{\varepsilon H_{r}R_{r}}{d_{0}^{2}}\sin\left(\Delta \theta_{n}\right)\\ b=a/C_{inp}\\ c=\left(1-3e_{\alpha }\right)\frac{1}{2}\frac{\varepsilon H_{r}R_{r}}{d_{0}^{3}}\Delta \theta_{n}\\ \mu =\cos^{2}\left(2\theta_{0}\right)\cos^{2}\left(\Delta \theta_{n}\right)+\frac{1}{3}\sin^{2}\left(2\theta_{0}\right)\sin^{2}\left(\Delta \theta_{n}\right)\\ \beta =\sin^{2}\left(2\theta_{0}\right)\cos^{2}\left(\Delta \theta_{n}\right)+\frac{1}{3}\cos^{2}\left(2\theta_{0}\right) \end{array}\right.$$

*Ke*_12_ and *Ke*_21_ representing in-pair modal coupling in terms of electrostatic stiffness are small compared to the mechanical stiffness and thus are neglected.

The *f*_1_, *f*_2_ terms contain the electrostatic actuation terms exciting the 2*θ* mode, and are derived from the electrostatic potential energy of the drive electrodes.

(A12) $$\left\{ \begin{array}{l} f_{1}=\left\{\begin{array}{l} \cos\left(2\theta_{0}\right)\left\{\left[1+2e_{d}\cos\left(4\phi_{w}\right)\left(1-2\beta \right)\right]v_{da}-\left[4e_{d}\sin\left(4\phi_{w}\right)\beta \right]v_{db}\right\}-\\ \sin\left(2\theta_{0}\right)\left\{-\left[4e_{d}\sin\left(4\phi_{w}\right)\mu \right]v_{da}-\left[1+2e_{d}\cos\left(4\phi_{w}\right)\left(1-2\mu \right)\right]v_{db}\right\} \end{array}\right\}aV_{p}\\ f_{2}=\left\{\begin{array}{l} \cos\left(2\theta_{0}\right)\left\{\left[4e_{d}\sin\left(4\phi_{w}\right)\beta \right]v_{da}-\left[1-2e_{d}\cos\left(4\phi_{w}\right)\left(1-2\beta \right)\right]v_{db}\right\}-\\ \sin\left(2\theta_{0}\right)\left\{\left[1-2e_{d}\cos\left(4\phi_{w}\right)\left(1-2\mu \right)\right]v_{da}-\left[4e_{d}\sin\left(4\phi_{w}\right)\mu \right]v_{db}\right\} \end{array}\right\}aV_{p} \end{array}\right.$$

The full expression of *e_r_*_1_, *e_r_*_2_, *e_sp_*_1_, and *e_sp_*_2_ in Equation (30) is shown as:

(A13) $$\left\{ \begin{array}{l} e_{sp1}=e_{4}\sin\left(4\theta_{sp}\right)\\ e_{sp2}=e_{4}\cos\left(4\theta_{sp}\right)\\ e_{r1}=8e_{1}e_{E}\sin\left(4\theta_{E4}\right)+4e_{2}\sin\left(4\theta_{w}\right)+2e_{w}^{2}e_{E}\sin\left(8\theta_{w}-4\theta_{E4}\right)\\ e_{r2}={8e}_{1}e_{E}\cos\left(4\theta_{E4}\right)+4e_{2}\cos\left(4\theta_{w}\right)+2e_{w}^{2}e_{E}\cos\left(8\theta_{w}-4\theta_{E4}\right) \end{array}\right.$$
